# MAPK, PI3K/Akt Pathways, and GSK-3β Activity in Severe Acute Heart Failure in Intensive Care Patients: An Updated Review

**DOI:** 10.3390/jcdd12070266

**Published:** 2025-07-10

**Authors:** Massimo Meco, Enrico Giustiniano, Fulvio Nisi, Pierluigi Zulli, Emiliano Agosteo

**Affiliations:** 1 Anesthesia and Intensive Care Department, San Carlo Clinic, Paderno Dugnano, 20030 Milan, Italy; emiliano.agosteo@clinicasancarlo.it; 2 Anesthesia and Intensive Care Department, Humanitas Hospital, Rozzano, 20089 Milan, Italy; enrico.giustiniano@humanitas.it (E.G.); fulvio.nisi@humanitas.it (F.N.); 3 Anesthesia and Intensive Care Department, European Hospital, Via Portuense 700, 00149 Rome, Italy; p.zulli@europeanhospital.it

**Keywords:** acute heart failure, MAPK signaling, PI3K/Akt pathway, GSK-3β, cardiomyocyte apoptosis, cardiac remodeling

## Abstract

Acute heart failure (AHF) is a clinical syndrome characterized by the sudden onset or rapid worsening of heart failure signs and symptoms, frequently triggered by myocardial ischemia, pressure overload, or cardiotoxic injury. A central component of its pathophysiology is the activation of intracellular signal transduction cascades that translate extracellular stress into cellular responses. Among these, the mitogen-activated protein kinase (MAPK) pathways have received considerable attention due to their roles in mediating inflammation, apoptosis, hypertrophy, and adverse cardiac remodeling. The canonical MAPK cascades—including extracellular signal-regulated kinases (ERK1/2), p38 MAPK, and c-Jun N-terminal kinases (JNK)—are activated by upstream stimuli such as angiotensin II (Ang II), aldosterone, endothelin-1 (ET-1), and sustained catecholamine release. Additionally, emerging evidence highlights the role of receptor-mediated signaling, cellular stress, and myeloid cell-driven coagulation events in linking MAPK activation to fibrotic remodeling following myocardial infarction. The phosphatidylinositol 3-kinase (PI3K)/Akt signaling cascade plays a central role in regulating cardiomyocyte survival, hypertrophy, energy metabolism, and inflammation. Activation of the PI3K/Akt pathway has been shown to confer cardioprotective effects by enhancing anti-apoptotic and pro-survival signaling; however, aberrant or sustained activation may contribute to maladaptive remodeling and progressive cardiac dysfunction. In the context of AHF, understanding the dual role of this pathway is crucial, as it functions both as a marker of compensatory adaptation and as a potential therapeutic target. Recent reviews and preclinical studies have linked PI3K/Akt activation with reduced myocardial apoptosis and attenuation of pro-inflammatory cascades that exacerbate heart failure. Among the multiple signaling pathways involved, glycogen synthase kinase-3β (GSK-3β) has emerged as a key regulator of apoptosis, inflammation, metabolic homeostasis, and cardiac remodeling. Recent studies underscore its dual function as both a negative regulator of pathological hypertrophy and a modulator of cell survival, making it a compelling therapeutic candidate in acute cardiac settings. While earlier investigations focused primarily on chronic heart failure and long-term remodeling, growing evidence now supports a critical role for GSK-3β dysregulation in acute myocardial stress and injury. This comprehensive review discusses recent advances in our understanding of the MAPK signaling pathway, the PI3K/Akt cascade, and GSK-3β activity in AHF, with a particular emphasis on mechanistic insights, preclinical models, and emerging therapeutic targets.

## 1. Introduction

Heart failure remains a major global health burden and represents one of the leading causes of hospitalization worldwide [1]. Acute heart failure (AHF) is defined by the sudden onset or rapid worsening of symptoms and may be triggered by a variety of clinical events, including acute myocardial infarction, hypertensive crises, or severe arrhythmias. AHF occurs as a consequence of an imbalance between the heart’s pumping ability and the body’s metabolic demands, involving mechanisms such as impaired myocardial contractility or relaxation, volume overload, elevated afterload, neurohormonal activation, and inflammatory and oxidative stress [2,3]. Acute heart failure (AHF) is characterized by a complex and dynamic interplay of pathophysiological mechanisms that rapidly compromise cardiac function and systemic perfusion. At the core of AHF lies a cascade of maladaptive responses involving hemodynamic stress, neurohormonal activation, inflammation, oxidative injury, and cellular dysfunction, which collectively accelerate myocardial deterioration and multi-organ involvement [4,5]. The initial insult—whether due to volume overload, pressure overload, or primary myocardial injury—leads to elevated left ventricular end-diastolic pressure (LVEDP) and impaired cardiac output. This acute increase in wall stress stimulates compensatory mechanisms that, while initially aimed at preserving circulatory stability, soon become deleterious. Prominent among these is the activation of neurohormonal systems, particularly the renin–angiotensin–aldosterone system (RAAS), the sympathetic nervous system, and arginine vasopressin release. These pathways induce systemic vasoconstriction, sodium and water retention, and tachycardia, thereby worsening the hemodynamic burden on the failing myocardium [6,7]. Concurrently, endothelial dysfunction contributes to increased vascular permeability, which facilitates fluid transudation into pulmonary and systemic tissues, manifesting as congestion and edema. This is compounded by the inflammatory milieu that characterizes AHF, with elevated levels of circulating cytokines such as tumor necrosis factor-α (TNF-α) and interleukins, which further impair vascular integrity and myocardial contractility [8]. Within the myocardium, a highly regulated network of intracellular signaling pathways responds dynamically to these stressors, mediating both adaptive and maladaptive changes in cardiac structure and function [9].

## 2. MAPK Pathway Family

**Figure 1:**
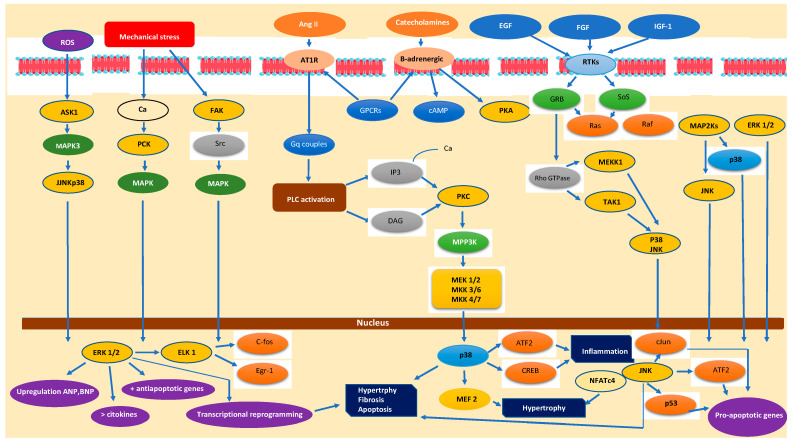
Upstreaming activation of MAPK signaling pathway in acute heart failure. Initiation via membrane receptors. (1) G Protein-Coupled Receptors (GPCRs): (A) Angiotensin II type I receptors (AT1R). Angiotensin II activates AT1R, which couples to Gq proteins. This triggers phospholipase C (PLC) activation, producing inositol triphosphate (IP3) and diacylglycerol (DAG), leading to calcium release and activation of Protein Kinase C (PKC). PKC can activate MAPK kinase kinases (MAP3Ks), such as Raf, initiating the MAPK cascade. (B) β-Adrenergic Receptors (β_1_, β_2_): catecholamines stimulate β-adrenergic receptors coupled to Gs proteins, increasing cAMP and PKA activity. PKA can modulate MAPK pathway components directly or indirectly, affecting ERK 1/2 activation. (2) Receptor Tyrosine Kinases (RTKs): growth factors such as EGF, FGF, and insulin-like growth factor (IGF-1) bind RTKs, causing receptor dimerization and autophosphorylation on tyrosine residues. These phosphorylated sites recruit adaptor proteins like Grb2 and SOS, facilitating the exchange of GDP for GTP on the small GTPase Ras, a crucial upstream activator of the MAPK cascade. (3) Small GTPases and Signal Propagation: (C) Ras Family: once GTP-bound, Ras activates the MAP3K Raf, which phosphorylates and activates MAP2Ks (MEK 1/2). This activation leads to the phosphorylation and activation of MAPKs (ERK 1/2). Rac and Rho GTPases: these regulate cytoskeletal dynamics and can activate other MAP3Ks like MEKK1 or TAK1, promoting activation of stress-activated MAPKs, such as p38 and JNK. (4) Mechanical Stress Sensors: (D) Integrins: mechanical overload causes conformational changes in integrins, which recruit and activate focal adhesion kinase (FAK). Activated FAK interacts with Src family kinases and small GTPases, linking mechanical signals to MAP3K activation, thus stimulating MAPK signaling. (E) Stretch-Activated Ion Channels: mechanical stretch can also increase intracellular calcium via stretch-sensitive channels, influencing kinase activation including PKC and calcium/calmodulin-dependent kinases that modulate MAPK activity. (5) Reactive Oxygen Species (ROS): acute stress increases mithochondrial ROS production. ROS can directly oxidize and modulate MAPK pathway components or upstream kinases such apoptosis signal-regulating kinase 1 (ASK1), a MAP3K that specifically activates JNK and p38 pathways, amplifying stress signaling. (6) Additional Upstream Kinases: (A) MAP3Ks (e.g., Raf, MEKK1, TAK1, ASK1): these act as convergence points for diverse upstream signals (GPCRs, RTKs, mechanical stress, ROS), phosphorylating and activating MAP2Ks. (B) MAP2Ks (e.g., MEK 1/2, MKK3/6, MKK4/7): these dual-specificity kinases phosphorylate threonine and tyrosine residues on MAPKs, finalizing their activation.

The MAPK family consists of serine/threonine kinases that are activated through sequential phosphorylation events within tightly regulated signaling cascades involving MAPK kinase kinases (MAP3Ks) and MAPK kinases (MAP2Ks). In the setting of acute heart failure (AHF), the rapid rise in circulating humoral mediators such as angiotensin II (Ang II), aldosterone, and endothelin-1 (ET-1) triggers the activation of MAPK pathways [10] (Figure 1). These cascades are not isolated; rather, they interact with other intracellular signaling networks—including Ras-Raf, PI3K-Akt, and various inflammatory pathways—further adding complexity to the intracellular signaling environment [11].

### 2.1. Canonical Architecture and Function

MAPK cascades are evolutionarily conserved signaling modules. The canonical MAPK pathway is typically composed of three hierarchical kinases: a MAP kinase kinase kinase (MAP3K), which phosphorylates and activates a MAP kinase kinase (MAP2K), which in turn phosphorylates the terminal MAP kinase (MAPK) isoforms on conserved threonine and tyrosine residues [12]. In the myocardium, the principal MAPK pathways involve ERK1/2, p38 MAPK, and JNK. ERK1/2 is primarily activated by growth factors and mechanical stretch, promoting cell survival and adaptive hypertrophy. In contrast, p38 and JNK are more responsive to stress stimuli and inflammatory cytokines, often triggering pro-apoptotic and pro-fibrotic responses [13]. The specificity of MAPK signaling is determined by its spatiotemporal dynamics; even subtle variations in the intensity or duration of activation can lead to distinct cellular outcomes. For instance, transient ERK1/2 activation may exert cardioprotective effects, whereas sustained ERK1/2 signaling contributes to maladaptive remodeling and progression to heart failure [14].

### 2.2. Upstream Activation in Acute Cardiac Stress

The mitogen-activated protein kinase (MAPK) pathways—including ERK1/2, JNK, and p38 MAPK—are central mediators of intracellular responses to extracellular stress signals. In acute heart failure (AHF), activation of MAPK cascades plays a critical role in driving maladaptive cardiac remodeling, cardiomyocyte apoptosis, fibrosis, and inflammation. A comprehensive understanding of the upstream triggers of MAPK activation is crucial for the development of novel therapeutic strategies. Acute heart failure (AHF) is driven by a variety of stressors that act as upstream activators of MAPK signaling. Neurohormones such as angiotensin II (AngII), aldosterone, and endothelin-1 (ET-1) bind to their respective receptors—typically G protein-coupled receptors (GPCRs) or receptor tyrosine kinases—on cardiomyocytes and cardiac fibroblasts, initiating intracellular signaling cascades that converge on MAPK pathways [15]. For example, AngII is a potent activator of both ERK1/2 and p38 MAPK, which not only mediate hemodynamic stress responses but also upregulate pro-inflammatory mediators, contributing to enhanced sympathetic drive and cardiomyocyte apoptosis [16]. Aldosterone, through both genomic and non-genomic mechanisms, has been shown to amplify MAPK signaling, thereby intensifying neurohormonal activation in central cardiovascular regulatory centers as well as in peripheral tissues [17]. ET-1, via its strong vasoconstrictive action, robustly activates p38 MAPK and JNK, promoting pro-fibrotic and inflammatory signaling cascades within the failing myocardium [18] (Figure 1). Cytokines such as tumor necrosis factor-alpha (TNF-α) and interleukin-1 beta (IL-1β) are elevated in acute heart failure (AHF) and contribute to the activation of JNK and p38 MAPK. These cytokines bind to their respective receptors—TNF receptor (TNFR) and interleukin-1 receptor (IL-1R)—initiating downstream signaling via TNF receptor-associated factors (TRAFs), and the kinase-transforming growth factor-β-activated kinase 1 (TAK1) [19]. Reactive oxygen species (ROS), generated during ischemia/reperfusion injury and mitochondrial dysfunction, also play a central role in MAPK activation. ROS function as second messengers by activating redox-sensitive kinases and membrane receptors such as Toll-like receptor 4 (TLR4) and epidermal growth factor receptor (EGFR), thereby amplifying MAPK signaling cascades [20]. Mechanical stress due to hemodynamic overload, as commonly observed in AHF, induces cardiomyocyte stretch and activates integrins and stretch-sensitive ion channels. These mechanosensors initiate MAPK signaling—particularly through ERK1/2—leading to the transcription of hypertrophic genes [21]. Angiotensin II type 1 (AT1) receptors, when activated by Ang II, couple to Gαq proteins and β-arrestins, triggering downstream activation of the Raf/MEK/ERK and p38 MAPK pathways [22]. Although β-adrenergic receptors are classically associated with cAMP/PKA signaling, they can also activate MAPK cascades indirectly through EGFR transactivation. Similarly, ligand binding to TNFR1 and IL-1R leads to TRAF recruitment and activation of MAP kinase kinase kinases (MAP3Ks), such as TAK1 [23]. TLR4, when engaged by pathogen- or damage-associated molecular patterns, activates both p38 and JNK MAPK pathways [24]. EGFR is frequently transactivated in cardiomyocytes through G protein-coupled receptor (GPCR) signaling or oxidative stress. Upon activation, EGFR promotes Ras-dependent ERK1/2 signaling, which contributes to either cardiomyocyte survival or pathological hypertrophy, depending on the context [25] (Figure 1).

### 2.3. Downstream Effects

#### 2.3.1. Inflammation

One of the key downstream effects of MAPK signaling is the regulation of inflammatory cytokine production. Activation of p38 MAPK and JNK in cardiomyocytes and cardiac fibroblasts enhances the transcription and secretion of pro-inflammatory cytokines such as tumor necrosis factor-alpha (TNF-α) and interleukin-1β (IL-1β) [26]. These cytokines act in a feed-forward manner to sustain cellular injury and further amplify MAPK activation. Additionally, the involvement of transcription factors such as nuclear factor-kappa B (NF-κB) and activator protein-1 (AP-1)—which are activated via MAPK-dependent phosphorylation—highlights the central role of MAPKs in orchestrating the inflammatory response [27]. A recent study [28] also demonstrated a mechanistic link between myeloid cell-derived coagulation signaling and the MAPK/TGF-β1 axis, emphasizing the contribution of MAPK pathways to fibrotic remodeling following ischemic injury.

#### 2.3.2. Apoptosis

MAPK pathways also play a critical role in regulating cardiomyocyte survival and death. Sustained activation of JNK and p38 MAPK during acute stress conditions promotes pro-apoptotic signaling in cardiomyocytes [29]. Apoptosis, along with necrosis, accelerates the loss of functional myocardial tissue, thereby contributing to the progression of heart failure. Experimental evidence indicates that pharmacological inhibition of these kinases reduces cardiomyocyte death and improves cardiac function [30]. For example, Min et al. [31] demonstrated that deficiency of MKP-5, a MAPK phosphatase and negative regulator of MAPK signaling, attenuates pressure overload-induced cardiac hypertrophy by modulating pathogenic kinase activity, suggesting that controlled regulation of MAPK signaling may confer cardioprotective effects.

#### 2.3.3. Fibrosis

The transition from acute myocardial injury to chronic heart failure often involves extensive fibrotic remodeling. MAPK cascades—particularly the p38 MAPK pathway—are key regulators of fibroblast activation, extracellular matrix (ECM) protein synthesis, and collagen deposition [32]. In response to MAPK activation, cardiac fibroblasts differentiate into myofibroblasts, which serve as the primary effectors of fibrotic scar formation [33]. Within cardiac fibroblasts, MAPK signaling critically drives phenotypic activation. ERK1/2 and p38 MAPK are strongly implicated in the transcriptional upregulation of genes encoding ECM proteins, including collagen types I and III, fibronectin, and periostin, thereby directly contributing to cardiac fibrosis and remodeling. JNK also plays a role, particularly in regulating fibroblast proliferation and apoptosis under stress conditions. Through these mechanisms, MAPK pathways control fibroblast-to-myofibroblast transformation, promote matrix protein synthesis, and facilitate pathological stiffening of the ventricular wall. Additionally, MAPK activation in fibroblasts enhances the secretion of pro-inflammatory cytokines and chemokines, which further recruit inflammatory cells and amplify local tissue injury. This feedback loop accelerates the progression from compensatory hypertrophy to maladaptive remodeling and pump dysfunction in acute heart failure [34]. Cardiac fibroblasts, when stimulated by stressors such as transforming growth factor-β1 (TGF-β1), angiotensin II (Ang II), and mechanical stretch, undergo transdifferentiation into myofibroblasts. These cells are characterized by the expression of α-smooth muscle actin (α-SMA) and the production of profibrotic proteins [35]. Among the MAPK pathways, p38 MAPK serves as a central mediator of myofibroblast differentiation and extracellular matrix (ECM) production. Activated by upstream kinases MKK3/6, p38 MAPK promotes the transcription of fibronectin (FN1) and other matrix components through phosphorylation of specific transcription factors. Pharmacological inhibition of p38 in rodent models of pressure overload or Ang II infusion significantly reduces myocardial fibrosis and improves cardiac function [36]. JNK is activated via MKK4/7 in response to oxidative and inflammatory stress. It phosphorylates c-Jun, facilitating AP-1-dependent transcription of profibrotic genes. JNK signaling also contributes to fibroblast proliferation and survival and is implicated in cardiomyocyte apoptosis [37]. ERK1/2, typically activated by mitogens such as fibroblast growth factor (FGF) and platelet-derived growth factor (PDGF), enhances fibroblast proliferation and migration through activation of ELK-1, and Ets-family transcription factors. While ERK1/2 also supports ECM protein expression, its role is more complex and may involve feedback regulation of TGF-β1 signaling [38].

#### 2.3.4. Crosstalk with Other Signaling Pathways

MAPK signaling in the heart does not function in isolation; rather, it is intricately integrated with other cellular signaling networks. One such example is the Ras–Raf–MEK–ERK cascade, in which receptor engagement activates Ras, leading to the phosphorylation of Raf and subsequent activation of ERK1/2 [39]. Crosstalk between MAPK and the PI3K–Akt pathways plays a pivotal role in determining the balance between hypertrophic growth and apoptosis. Additionally, recent studies have highlighted the involvement of Toll-like receptor (TLR) signaling and nuclear factor-kappa B (NF-κB) activation in mediating inflammatory responses in parallel with MAPK cascades. Duangrat et al. [40] reported that activation of A3 adenosine receptors modulates MAPK signaling, conferring cardioprotective effects. Emerging evidence also suggests that mitochondrial dysfunction and oxidative stress serve both as upstream activators and downstream consequences of MAPK activation. For example, p47^phox-dependent ROS signaling through apoptosis signal-regulating kinase 1 (ASK1) and MKK3/6 has been shown to activate MAPK pathways in response to angiotensin II infusion, thereby linking oxidative stress to cardiac hypertrophy and apoptosis [41]. This bidirectional interaction underscores the complexity of MAPK regulation in cardiomyocyte survival under stress conditions.

## 3. PI3K Pathway

**Figure 2:**
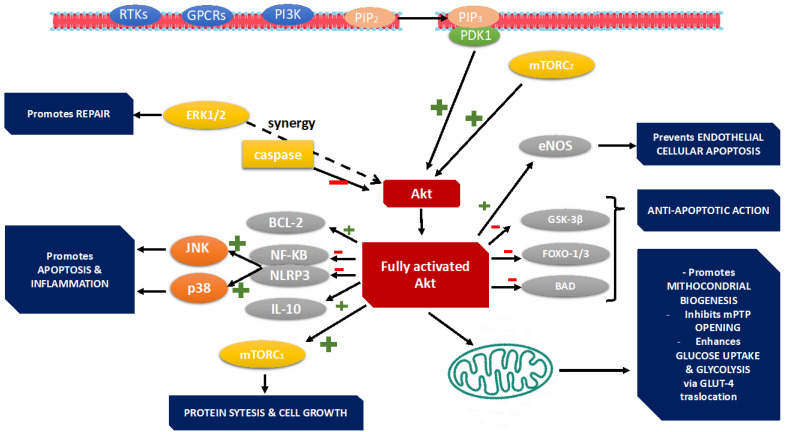
PI3K/Akt pathway activation in acute heart failure. PI3Ks are divided into: (a) Class I (most relevant in heart), subdivided into IA (PI3Kα, PI3Kβ, PI3Kδ) and IB (PI3Kγ), and (b) Class II and III: less relevant in cardiac signaling. PI3Kα is activated by receptor tyrosine kinases (RTKs) such as IGF-1, insulin, and neuregulins. PI3Kγ is activated by G protein-coupled receptors (GPCRs) such as those for Ag II, catecholamines, and ET-1. Ligand binding activates RTKs or GPCRs, and PI3K phosphorylates PIP2 → PIP3. PDK1 and Akt are recruited to the membrane. Akt is activated by PDK1 and by mTORC2. Activated Akt phosphorylates multiple downstream substrates. (1) Cardiomyocyte Survival: inhibits apoptosis via phosphorylation of BAD (Bcl-2-associated death promoter), FOXO transcription factors, and GSK-3β, which regulates mitochondrial integrity. Promotes anti-apoptotic proteins: Bcl-2, Bcl-xL. Suppresses mitochondrial cytochrome c release and caspase-9 activation. (2) Mitochondrial Function. Increases hexokinase-II binding to mitochondria, preserving membrane potential. Enhances ATP production and glucose metabolism. Activates PGC-1α, promoting mitochondrial biogenesis. Prevents mitochondrial permeability transition pore (mPTP) opening. (3) Endothelial and Vascular Protection. Phosphorylates and activates eNOS, maintains endothelial barrier integrity, improves coronary microcirculatory function in AHF. (4) Inflammatory Modulation. Inhibits NF-κB, NLRP3 inflammasome, and proinflammatory cytokines (IL-6, TNF-α), and promotes IL-10 and M2 macrophage polarization. Prevents leukocyte–endothelium adhesion via VCAM-1 and ICAM-1 suppression. (5) Crosstalk with other signaling pathways. PI3K/Akt and GSK-3β Akt phosphorylate and inactivate GSK-3β. Prevent apoptosis and preserve mitochondrial function. Regulate hypertrophic responses. PI3K/Akt and MAPK MAPKs (ERK, JNK, p38) are activated in AHF and interact with Akt: ERK may synergize with Akt in survival. JNK/p38 may oppose PI3K/Akt by promoting inflammation and apoptosis. Crosstalk determines outcome: survival vs. remodeling PI3K/Akt and mTOR. + is activation; − is inhibition.

The PI3K/Akt pathway is activated downstream of receptor tyrosine kinases (RTKs) or G protein-coupled receptors (GPCRs) in response to a variety of extracellular stimuli, including growth factors, cytokines, and mechanical stress [42] (Figure 2). Among the PI3K family, Class I isoforms—particularly PI3Kα, PI3Kβ, and PI3Kγ—are the most extensively studied in cardiovascular research. PI3Kα, which is activated by RTKs such as the insulin-like growth factor-1 receptor (IGF-1R), promotes physiological hypertrophy, enhances myocardial contractility, and supports metabolic function through downstream activation of Akt. In contrast, PI3Kγ is primarily activated via GPCRs and is involved in regulating inflammatory responses and β-adrenergic signaling, often contributing to maladaptive remodeling under chronic stress conditions [43]. PI3Kβ, though less well characterized in the heart, may function as part of compensatory signaling during stress. In acute heart failure (AHF), stressors such as ischemia–reperfusion injury, sepsis, or toxic insults lead to cardiomyocyte apoptosis, metabolic dysregulation, and inflammation. The PI3K–Akt–mTOR axis plays a central role in modulating these pathological processes: (A) Cardiomyocyte survival: Akt activation inhibits apoptosis by suppressing pro-apoptotic factors (e.g., Bad, GSK-3β) and promoting survival signals (e.g., Bcl-2). (B) Inflammation: PI3Kγ facilitates leukocyte recruitment and cytokine production, worsening myocardial injury in settings like sepsis and myocarditis. (C) Contractile function: PI3Kα improves calcium handling and excitation–contraction coupling, both of which are impaired in AHF [43]. Among the Class I isoforms, PI3Kα plays a particularly cardioprotective and pro-contractile role. It is predominantly activated downstream of RTKs such as IGF-1R and the insulin receptor (IR), triggering the PI3K–Akt–mTOR signaling cascade [44]. In the healthy myocardium, PI3Kα supports physiological hypertrophy, sustains contractility, and maintains metabolic homeostasis. Targeted activation of this isoform represents a promising therapeutic approach to mitigate the functional decline associated with acute cardiac decompensation. Importantly, PI3Kα signaling is crucial for the regulation of intracellular calcium homeostasis—an essential component of excitation–contraction coupling. Activation of the PI3Kα–Akt axis promotes phosphorylation of phospholamban (PLB), enhancing the activity of sarcoplasmic reticulum Ca^2+^-ATPase (SERCA2a) and facilitating calcium reuptake into the sarcoplasmic reticulum. This mechanism contributes to improved myocardial relaxation and contractile performance, both of which are significantly impaired in AHF. In transgenic mouse models, overexpression of a constitutively active PI3Kα subunit leads to increased ejection fraction, stroke volume, and cardiac output without inducing pathological hypertrophy [45]. These findings suggest that transient or acute upregulation of PI3Kα may effectively counteract the contractile dysfunction that characterizes AHF. Akt translocates into the nucleus, promotes transcription of survival, metabolic, and growth-related genes, enhances glucose uptake and glycolysis, and increases fatty acid oxidation and mitochondrial biogenesis. Akt activates mTOR (mechanistic Target of Rapamycin): mTOR further supports protein synthesis, cell growth, and hypertrophy [46]. Activation of the PI3K/Akt signaling pathway in the heart initiates a broad spectrum of adaptive responses that collectively enhance myocardial performance and resilience during stress. One of the primary outcomes is improved metabolic function. PI3K/Akt activation promotes glucose uptake through GLUT4 translocation and enhances glycolysis, thereby increasing ATP production to meet the high energy demands of stressed or failing cardiomyocytes. Additionally, it supports mitochondrial function and biogenesis, which are essential for maintaining cardiac energetics, particularly during acute ischemic events. Beyond metabolic regulation, the pathway plays a critical role in promoting cardiomyocyte survival. Akt inhibits key components of the apoptotic machinery, including BAD and caspase-9, while simultaneously enhancing the expression of anti-apoptotic proteins such as Bcl-2. These effects reduce myocardial cell death in conditions such as ischemia–reperfusion injury or sepsis-induced cardiomyopathy, thereby preserving myocardial integrity and function. Another crucial effect of PI3K/Akt signaling is its role in regulating myocardial growth. In physiological contexts, such as during exercise or pregnancy, activation of this pathway induces controlled cardiomyocyte hypertrophy. This form of hypertrophy is characterized by proportional increases in cell size, preserved sarcomeric organization, and improved function, in contrast to the maladaptive remodeling observed in pathological states [47].

### 3.1. Cardiomyocyte Survival and Apoptosis

Cardiomyocyte loss through programmed cell death is a central feature in the development of acute heart failure. Activation of the PI3K/Akt pathway inhibits apoptotic signaling by phosphorylating and inactivating key pro-apoptotic proteins, such as caspase-9, and by upregulating anti-apoptotic molecules including Bcl-2 [48].

Studies in animal models of acute myocardial injury have revealed that timely activation of Akt reduces infarct size and preserves left ventricular function by suppressing apoptotic cascades [49].

Moreover, the cardioprotective effects of nicorandil in diabetic cardiomyopathy have been directly linked to enhanced Akt phosphorylation, which leads to increased nitric oxide (NO) production through eNOS activation [50].

Collectively, these studies suggest that PI3K/Akt activation represents an adaptive response that confers resistance to stress-induced apoptosis and may serve as a critical determinant of myocardial viability during acute stress episodes [51].

### 3.2. Regulation of Hypertrophy

Adaptive hypertrophy is initially beneficial in increasing cardiac output; however, prolonged hypertrophy becomes maladaptive and contributes to heart failure.

The PI3K/Akt pathway is central to this process, mediating both physiologic and pathologic aspects of cardiac hypertrophy.

Early activation of Akt promotes protein synthesis through mTOR activation and enhances cardiomyocyte growth in response to increased workload. Nonetheless, sustained Akt activation may lead to pathological remodeling, characterized by fibrosis, increased cardiomyocyte size, and eventual contractile dysfunction [52].

Recent evidence from pressure overload-induced heart failure models indicates that pharmacological agents such as carnosol not only prevent ventricular arrhythmias but also mitigate adverse remodeling by modulating downstream targets of Akt, including Sirt1 and GSK-3β [53].

In addition, microRNAs (miRNAs) have emerged as key regulators of Akt-mediated hypertrophy. For example, alterations in the expression of miR-132 and miR-146 have been implicated in controlling hypertrophic pathways by modulating PTEN and indirectly influencing Akt activity. These findings highlight the necessity for precise modulation of Akt signaling—a therapeutic strategy that may preserve the beneficial adaptations while forestalling maladaptive remodeling [54].

### 3.3. Mitochondrial Function

Cardiac contractility and energy homeostasis are inextricably linked to mitochondrial function, and the PI3K/Akt pathway plays a significant role in this regulation. Upon activation, Akt influences the metabolic state of cardiomyocytes by modulating glucose uptake, lipid metabolism, and mitochondrial biogenesis.

Akt-mediated phosphorylation of key metabolic regulators improves mitochondrial efficiency and prevents metabolic derangements that exacerbate ischemic injury. Recent work has demonstrated that Akt activation enhances the phosphorylation of pyruvate dehydrogenase kinases and mitochondrial transcription factors, thereby reducing reactive oxygen species (ROS) production and improving ATP synthesis during acute cardiac insult [55]. These metabolic benefits are particularly vital in the setting of acute heart failure, where energetic deficits and oxidative stress converge to promote myocardial dysfunction. Taken together, the ability of PI3K/Akt to coordinate metabolic adaptation reinforces its potential as a target for interventions aimed at optimizing mitochondrial function and overall cardiac energetics during heart failure episodes.

### 3.4. Inflammation, Oxidative Stress, and Autophagy

Acute heart failure is frequently associated with heightened inflammatory responses and oxidative stress, both of which contribute to ongoing myocardial injury. The PI3K/Akt pathway has been shown to exert potent anti-inflammatory effects by interfering with the activation of nuclear factor-kappa B (NF-κB), and related cytokine cascades. In experimental models, Akt activation correlates with reduced expression of TNF-α, interleukins, and other pro-inflammatory mediators that can otherwise exacerbate myocyte damage. Furthermore, PI3K/Akt signaling is intricately involved in regulating autophagy, a cellular process that recycles damaged proteins and organelles during stress [56].

Controlled autophagy may remove dysfunctional mitochondria and proteins in acutely injured myocardium, thereby mitigating cell death and preserving contractile function. However, excessive or dysregulated autophagy can be detrimental, underscoring the importance of a balanced modulation of this pathway. Recent studies have even shown that modulation of the microRNA/PI3K/Akt axis in doxorubicin-induced cardiotoxicity can normalize both autophagic flux and apoptotic responses, providing further evidence for the therapeutic benefit of targeting this cascade in acute cardiac injury [57].

## 4. GSK-3β Pathway

**Figure 3:**
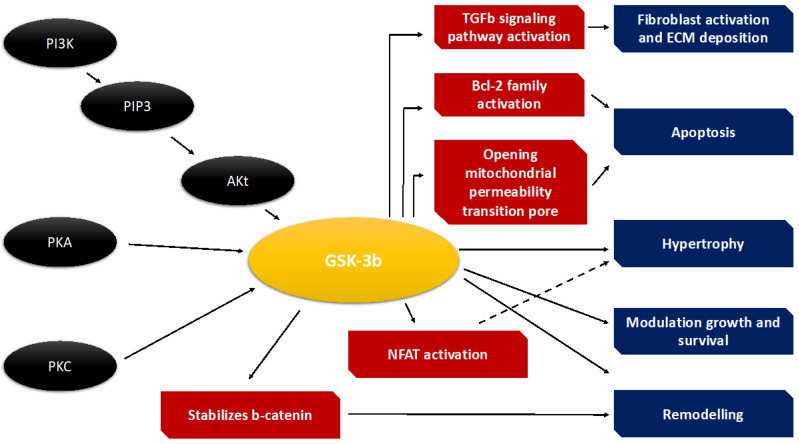
The figure illustrates the GSK-3β (Glycogen Synthase Kinase-3 beta) signaling pathway in the heart, highlighting its upstream activators and downstream effects on cardiac cell fate and function. Receptors and upstream activation: G protein-coupled receptors (GPCRs), Tyrosine kinase receptors (e.g., IGF-1R, insulin receptor), Integrins. These receptors activate PI3K (phosphoinositide 3-kinase) upon binding of ligands such as growth factors, cytokines, or mechanical stimuli. PI3K → AKT Pathway: activated PI3K catalyzes the formation of PIP3, which recruits and activates AKT (also known as Protein Kinase B). AKT phosphorylates GSK-3β, inhibiting its activity. GSK-3β Downstream effects: when GSK-3β is inactive (phosphorylated): promotes cardiomyocyte survival, facilitates cardiac hypertrophy as an adaptive mechanism. When GSK-3β is active (not phosphorylated): leads to mitochondrial dysfunction, triggers cardiomyocyte apoptosis or cell death. Adaptive or maladaptive remodeling, depending on the balance between cell survival, hypertrophy, and death. Clinical implications: overactive GSK-3β has been implicated in heart failure, myocardial infarction, and pathological hypertrophy. Inhibition of GSK-3β is considered cardioprotective in preclinical models and is a potential therapeutic target.

GSK-3β is a serine/threonine kinase that was originally identified in the context of glycogen metabolism. Its activity is regulated by phosphorylation at key residues, with phosphorylation at Ser9 leading to inactivation and phosphorylation at Tyr216 (pY216) being associated with its active state [58]. The enzyme has a ubiquitous expression profile and is involved in a wide range of cellular processes including cell cycle regulation, transcription, and apoptosis [59] (Figure 3).

Recent work has advanced the understanding of noncanonical regulatory mechanisms, such as S-nitrosylation (SNO), which can control GSK-3β activity independently of its phosphorylation status. In a study by Wang et al. [60], conditional SNO modification of GSK-3β was shown to affect its subcellular localization in cardiomyocytes, thereby modulating its downstream effectors during the development of heart failure. Additionally, novel insights into its regulation by post-translational modifications—including O-GlcNAcylation—suggest that increased O-GlcNAc levels under stress may hinder its inactivation and thus exacerbate cardiac dysfunction. Beyond these modifications, emerging data also implicate upstream kinases and phosphatases, such as Akt and protein phosphatase 2A, in fine-tuning GSK-3β activity under conditions of acute stress [61].

### 4.1. GSK-3β in the Pathogenesis of Acute Heart Failure

During acute cardiac injury, the heart undergoes rapid remodeling, a process that involves myocyte hypertrophy, fibrosis, and subsequent deterioration of contractile function. Traditionally, GSK-3β has been characterized as a negative regulator of cardiac hypertrophy, as its inactivation is required for growth-promoting signals following stress [62]. Human data suggest that downregulation of GSK-3β in the setting of hypertrophy may serve as a compensatory mechanism in early heart failure stages; however, prolonged suppression can lead to maladaptive remodeling and scar formation. Recent experimental studies in rodent models, such as those by Tariq et al. [63], have demonstrated that pharmacological modulation of GSK-3β can have differential effects on hypertrophy. Additional work has shown that targeting GSK-3β can alter the expression of key transcription factors, such as NFAT, thereby regulating the hypertrophic response [6]. One of the hallmark features of acute heart failure is enhanced cardiomyocyte apoptosis that leads to deteriorating cardiac function. GSK-3β is centrally implicated in apoptotic signaling cascades, where its activation promotes the phosphorylation of downstream targets that enhance cell death. The kinase has been shown to influence apoptosis through several mechanisms, such as the regulation of mitochondrial permeability transition pore (mPTP) opening, modulation of Bcl-2 family proteins, and activating pro-apoptotic transcription factors [64]. Recent studies indicate that SNO of GSK-3β [65,66] alters its mitochondrial localization and influences apoptotic outcomes. Thus, the balance between active and inactive GSK-3β is crucial in determining the survival of cardiomyocytes during acute stress.

Acute injury in the myocardium is accompanied by complex inflammatory responses that lead to fibrosis, further impairing cardiac function. GSK-3β is a significant modulator of the inflammatory cascade via its regulation of nuclear factor-kappa B (NF-κB) activity and cytokine expression [67].

Ding et al. [68] reported that in models of caerulein-induced acute pancreatitis, GSK-3β-mediated activation of NF-κB resulted in upregulation of pro-inflammatory cytokines. In the heart, similar pathways contribute to the progression of AHF. Persistent activation of GSK-3β has been linked to increased production of IL-6 and TNF-α, promoting an environment that favors fibrotic deposition and adverse remodeling. In addition, studies have shown that inhibition of GSK-3β can attenuate transforming growth factor-beta (TGF-β)-induced fibroblast-to-myofibroblast conversion, thereby reducing collagen deposition and fibrosis in models of acute cardiac injury. These data suggest that therapeutic modulation of GSK-3β could provide dual benefits by reducing both the inflammatory response and subsequent fibrotic remodeling in AHF [69,70].

Metabolic disturbances, including lipotoxicity, are major contributors to cellular damage in heart failure. Emerging evidence indicates that GSK-3β is instrumental in modulating lipid metabolism within the heart, especially under conditions of high fat stress [71], and elucidated that in obesity-induced cardiomyopathy, activation of GSK-3β dampens the expression of adipose triglyceride lipase (ATGL) through post-translational regulation of FoxO1. This leads to lipid accumulation and exacerbates lipotoxicity in the myocardium. Furthermore, experimental deletion of cardiac-specific GSK-3β in murine studies has been correlated with altered expression of lipid handling proteins, suggesting that precise modulation of GSK-3β activity is necessary to maintain myocardial lipid homeostasis during acute injury. Metabolic reprogramming also involves a complex interplay with mitochondrial function, where GSK-3β’s regulation of mitochondrial dynamics and mitophagy plays a critical role in cardioprotection [72].

### 4.2. GSK-3β Dysregulation in Acute Heart Failure

Recent research has also focused on the role of microRNAs (miRNAs) and epigenetic modifications in the regulation of GSK-3β expression and activity. Dysregulation of specific miRNAs can lead to abnormal GSK-3β levels, which in turn contribute to adverse cardiac remodeling. For example, alterations in miR-132 and miR-146 expression have been associated with modulation of PTEN, thereby indirectly influencing GSK-3β activity. In parallel, epigenetic modifications, such as histone acetylation and methylation, have been shown to affect the transcription of the GSK-3β gene under stress conditions. These molecular-level insights offer additional avenues for therapeutic intervention, including the development of miRNA mimics or inhibitors designed to fine-tune GSK-3β activity in acute heart failure. Recent studies have underscored that GSK-3β activity is finely regulated by a range of post-translational modifications (PTMs) beyond the classical phosphorylation-dependent mechanisms [73]. Among these, S-nitrosylation (SNO) has emerged as a crucial regulatory layer, influencing the kinase’s subcellular localization and substrate specificity. Likewise, O-GlcNAcylation has been shown to induce aberrant GSK-3β activation under pressure overload, thereby impairing compensatory hypertrophic responses in cardiomyocytes [74]. These modifications critically determine the functional output of GSK-3β signaling in the stressed myocardium. In addition, emerging proteomic analyses have identified novel PTMs that modulate GSK-3β’s interaction with its substrates, further shaping downstream effects on cell survival, fibrosis, and remodeling outcomes [75] (Figure 3). The pathological role of GSK-3β in acute heart failure (AHF) is further amplified by its dynamic crosstalk with other key signaling pathways. Substantial evidence indicates that GSK-3β negatively regulates hypertrophic signaling through modulation of nuclear factor of activated T-cell (NFAT) activity and its interaction with the Akt/mTOR axis [76]. In this regulatory network, inhibition of Akt leads to enhanced GSK-3β activity, suppressing hypertrophic gene transcription. Conversely, Akt-mediated phosphorylation of GSK-3β results in its inactivation, thereby promoting adaptive cardiomyocyte growth. This intricate balance reveals the therapeutic potential of dual-targeting strategies aimed at simultaneously modulating both Akt and GSK-3β to mitigate maladaptive remodeling while preserving pro-survival signaling. Additional studies have shown that GSK-3β interfaces with the RAF-MEK-ERK pathway in cardiac fibroblasts, promoting fibrotic remodeling in response to injury. Targeting this intersection may allow for the development of combinatorial therapies that inhibit both apoptosis and fibrosis [77]. More recently, attention has turned to the epigenetic and microRNA (miRNA)-based regulation of GSK-3β expression and activity [78]. Dysregulation of specific miRNAs alters GSK-3β levels and activity, thereby contributing to adverse structural and functional changes in the myocardium. For example, miR-132 and miR-146 modulate PTEN expression, which in turn influences the PI3K/Akt/GSK-3β axis. In parallel, epigenetic modifications—including histone acetylation and methylation—have been shown to affect GSK-3β gene transcription during cardiac stress. Together, these findings unveil novel therapeutic opportunities, such as the use of miRNA mimics or inhibitors, or epigenetic modulators, aimed at fine-tuning GSK-3β activity to prevent adverse remodeling and improve cardiac function in AHF [79,80].

## 5. MAPK, PI3K/Akt, and GSK-3β Signaling in Acute Heart Failure in Diabetic Patients

Diabetes mellitus (DM) dramatically worsens outcomes in acute heart failure (AHF), amplifying myocardial injury, metabolic dysfunction, and inflammatory stress. At the molecular level, intracellular signaling cascades—particularly the mitogen-activated protein kinase (MAPK), phosphoinositide 3-kinase/protein kinase B (PI3K/Akt), and glycogen synthase kinase-3β (GSK-3β) pathways—are deeply altered by hyperglycemia and insulin resistance, driving maladaptive cardiac remodeling and contractile dysfunction [81].

### 5.1. MAPK Mechanisms and Activation

The MAPK family respond to diabetic stressors such as reactive oxygen species, cytokines, and advanced glycation end-products (AGEs) [82]. These kinases are activated through a hierarchical phosphorylation cascade, and their regulation is exaggerated in the diabetic myocardium. ERK1/2 is involved in hypertrophic signaling. While initially adaptive, persistent activation promotes pathological hypertrophy in diabetic AHF [83] p38. MAPK is strongly upregulated in diabetic hearts, driving pro-fibrotic gene expression, mitochondrial dysfunction, and insulin resistance [84]. JNK is activated by hyperglycemia and free fatty acids, promoting cardiomyocyte apoptosis and interfering with insulin receptor substrate-1 (IRS-1) signaling, thus suppressing PI3K/Akt activity.

### 5.2. PI3K/Akt Mechanisms and Activation

In diabetic hearts, insulin resistance leads to impaired IRS-1 signaling, reduced PI3Kα activity, and deficient Akt phosphorylation [85]. Simultaneously, PI3Kγ is hyperactivated due to chronic β-adrenergic stimulation and inflammation. Reduced Akt activity compromises glucose uptake, mitochondrial function, and anti-apoptotic signaling. PI3Kγ activation exacerbates inflammation, fibrosis, and contractile dysfunction through PKA/β-arrestin coupling and NF-κB activation. Downstream effectors such as mTOR are also disrupted, impairing autophagy and exacerbating myocardial stress [86].

### 5.3. GSK-3β Mechanisms and Activation

GSK-3β, a downstream target of Akt, remains hyperactive in diabetic hearts due to reduced Akt-mediated inhibitory phosphorylation. Active GSK-3β enhances apoptosis and interferes with glycogen synthesis, exacerbating metabolic derangements, and promotes cardiomyocyte apoptosis by regulating mitochondrial permeability and nuclear transcription factors (e.g., NFAT). It also contributes to contractile dysfunction and fibrotic remodeling and interferes with insulin-mediated glycogen synthesis, worsening energy deficits [87].

## 6. Therapeutic Perspectives

### 6.1. MAPK Pathway

Given its central role in maladaptive remodeling, the MAPK pathway has emerged as a compelling therapeutic target in AHF. Experimental inhibition of p38 MAPK, for instance, has been shown to reduce inflammation, fibrosis, and myocardial cell death in preclinical models [88]. Similarly, inhibition of JNK activity can attenuate pathological hypertrophy and improve ventricular function [89].

ERK1/2, although generally associated with adaptive hypertrophy, may also contribute to pathological remodeling under sustained activation, suggesting context-dependent roles [90]. The rationale for MAPK inhibition is strengthened by the observation that each arm of the pathway plays a role in different aspects of cardiac pathology. p38 MAPK is strongly associated with the transcription of pro-inflammatory and profibrotic genes, while JNK has been linked to cardiomyocyte apoptosis and maladaptive remodeling. ERK1/2 signaling, while protective in short-term stress responses, can promote hypertrophy and fibrosis if dysregulated. Targeting MAPK signaling poses several challenges. The ubiquity and pleiotropic effects of these kinases mean that broad inhibition can result in off-target toxicity, including impairments in immune function and tissue repair. Nevertheless, the development of isoform-specific inhibitors (e.g., p38α-selective compounds) and pathway-restricted molecules (e.g., MEK1/2 inhibitors) is advancing.

Losmapimod, a selective p38 MAPK inhibitor, demonstrated antifibrotic and anti-inflammatory effects in preclinical studies and entered clinical evaluation for heart failure and myocardial infarction, although trials such as SOLSTICE yielded mixed results in terms of clinical outcomes [91]. Moreover, therapeutic agents already in clinical use—such as angiotensin receptor blockers (ARBs), beta-blockers, and mineralocorticoid receptor antagonists—may exert beneficial effects by indirectly modulating MAPK signaling. For instance, ARBs can suppress Ang II-mediated activation of ERK1/2 and p38 MAPK, reducing downstream oxidative stress and inflammation [92]. Beta-blockers may also impact MAPK signaling by reducing adrenergic drive and associated receptor cross-talk. Emerging interest also surrounds upstream modulators such as Toll-like receptor 4 (TLR4), epidermal growth factor receptor (EGFR), and β-arrestin signaling complexes. β1-adrenergic receptor transactivation of EGFR via β-arrestin scaffolding has been shown to activate cardioprotective ERK signaling, suggesting that biased ligands or pathway-specific agonists might selectively harness beneficial aspects of MAPK signaling [93].

Future strategies may also involve dual-pathway modulation, targeting MAPK in conjunction with PI3K/Akt, or NF-κB pathways to provide synergistic benefit. Furthermore, biomarker-guided stratification of patients based on inflammatory or fibrotic phenotypes may improve the success of MAPK-targeted therapies (Table 1).

### 6.2. PI3K/Akt/mTor Signaling Cascade

Targeting the PI3K pathway offers a promising strategy to modulate multiple pathological features of AHF simultaneously. Three main PI3K isoforms are expressed in the cardiovascular system: PI3Kα, PI3Kβ, and PI3Kγ, each with distinct roles.

PI3Kγ inhibitors, such as IPI-549 and AS-605240, selectively inhibit PI3Kγ, reduce inflammation, and preserve β-adrenergic signaling.

Preclinical evidence shows reduced cardiac injury in myocarditis and sepsis-induced cardiomyopathy [94]. PI3Kα agonists may enhance contractility and metabolic support but pose a potential oncogenic risk with chronic activation. No direct clinical agonist is currently available; however, gene therapy trials have explored PI3Kα upregulation [95].

SC79 and MK-2206 (Akt modulators) promote cardiomyocyte survival and reduce apoptosis in ischemia–reperfusion and pressure overload models [96,97]. These agents enhance glucose uptake and preserve mitochondrial integrity.

Rapamycin, also known as sirolimus, is a macrolide compound that forms a complex with FKBP12 to inhibit mTOR complex 1 (mTORC1). This leads to the suppression of downstream signaling through S6K1 and 4EBP1, thereby attenuating protein synthesis, cell growth, and pathological cardiac hypertrophy [98].

Perino et al. [99] showed that genetic deletion of PI3Kγ in mice improves survival in models of heart failure by reducing inflammation and preserving β-adrenergic signaling. Zhao et al. [100] demonstrated that Akt activation protects against ischemia-induced apoptosis in cardiomyocytes. Shende et al. [101] found that mTOR inhibition ameliorates maladaptive hypertrophy but must be tightly regulated (Table 1).

### 6.3. GSK-3β Pathway

Dysregulated GSK-3β pathway activity contributes to cardiomyocyte death, maladaptive remodeling, and impaired contractile recovery. Recent preclinical evidence highlights the cardioprotective potential of selective GSK-3β inhibition during acute decompensated states. Pharmacologic inhibition of GSK-3β has been extensively explored in preclinical models.

Pharmacological inhibition of GSK-3β has been shown to protect against ischemia–reperfusion (I/R) injury in both rodent and porcine hearts. Inhibition limits mitochondrial permeability transition pore (mPTP) opening, reduces myocardial cell death, and decreases infarct size [102].

Cardiac-restricted expression of a dominant negative GSK-3β mutant (Tg-GSK-3β-DN) in mice—driven by the α-MHC promoter—results in less apoptosis, fibrosis, and pulmonary congestion following transverse aortic constriction (TAC). In contrast, mice expressing constitutively active GSK-3β resist hypertrophic remodeling initially but eventually progress to systolic failure under persistent pressure stress [103].

In failing hearts, GSK-3β is chronically inactivated (via Ser9 phosphorylation) by upstream kinases such as Akt and PKA, particularly under adrenergic and neurohumoral stress—removing the brake on hypertrophy, fibrosis, and metabolic decompensation.

Lithium inhibits GSK-3β through two mechanisms. First, it acts as a direct inhibitor by competing with magnesium (Mg^2+^) at the kinase’s catalytic site. This reduces the enzyme’s catalytic efficiency without competing for ATP, a non-competitive mode of action. Second, lithium indirectly suppresses GSK-3β by increasing the activity of upstream kinases such as Akt and PKA, which phosphorylate GSK-3β at the inhibitory Ser9 residue [104].

In rodent and porcine models of ischemia–reperfusion injury, lithium administration before or during ischemia significantly reduces infarct size by preventing the opening of the mitochondrial permeability transition pore (mPTP), an event mediated by GSK-3β-dependent pathways. Gross et al. [105] showed that lithium-treated rat hearts subjected to coronary occlusion had a 30–50% reduction in infarct size, attributed to decreased mitochondrial dysfunction and reduced apoptotic signaling. Similarly, in hypertrophied rabbit hearts, pre-treatment with lithium improved recovery of left ventricular developed pressure and preserved diastolic relaxation, indicating a cardioprotective role during acute stress [106].

While lithium’s cardioprotective effects are well established in animal models, its translation into cardiovascular therapeutics faces several hurdles. One major limitation is its non-selectivity for GSK-3 isoforms. GSK-3α has cardioprotective roles, particularly in limiting fibrosis and preserving systolic function. Broad inhibition of both isoforms may swap one pathology (e.g., hypertrophy) for another (e.g., fibrosis). This has prompted the development of newer GSK-3β-selective compounds such as tideglusib and 9-ING-41, which are currently being evaluated in oncology and neurology but remain untested in heart failure. The second challenge is the need for temporal precision. GSK-3β activity is beneficial during chronic overload (to limit hypertrophy), but its acute inhibition during ischemic episodes (e.g., MI, cardiac surgery) may provide preconditioning-like benefits. Thus, smart delivery systems or gene circuits that enable temporal modulation of GSK-3β could optimize therapeutic outcomes [107]. Third, safety concerns regarding lithium’s pro-arrhythmic potential must be addressed through dedicated cardiac safety screens. Induced pluripotent stem cell-derived cardiomyocytes (iPSC-CMs), patch clamp assays, and telemetry in large animal models are essential to evaluate conduction effects prior to human trials.

Tideglusib is a GSK-3β–specific inhibitor tested in Alzheimer’s and oncology. While not yet trialed in heart failure, early-phase data show no major cardiac toxicity. SB216763 inhibits GSK-3β potently and reversibly. However, in ex vivo human cardiac slices, it was shown to reduce conduction velocity and decrease Nav1.5 expression, suggesting potential pro-arrhythmic risk [108] (Table 1).

## Figures and Tables

**Table 1:** Therapeutic targeting of MAPK, PI3K/Akt, and GSK-3β pathways in acute heart failure.

Pathway	Targeted Molecule	Mechanism of Action	Therapeutic Agents	Potential Benefit	Limitation/Risks
MAPK	p38, ERK 1/2, JNK	Inhibition of stress-activated kinases reduces inflammation, fibrosis, hypertrophy, and cardiomyocyte apoptosis	SB203580 (p38 inhibitor), SP600125 (JNK inhibitor), U0126 (ERK1/2 inhibitor)	Decreases maladaptive cardiac remodeling, improves ventricular function	Non-specific inhibition may impair physiological adaptation to stress
PI3K/Akt	PI3Kγ, PI3Kα, Akt1	Activation enhances cardiomyocyte survival, mitochondrial metabolism, and contractility; reduces apoptosis	SC79 (Akt activator), MK-2206 (Akt inhibitor), IPI-549 (PI3Kγ inhibitor)	Improves energy efficiency and cell viability; supports adaptive hypertrophy	Oncogenic potential (especially PI3Kα); context- and dose-dependent responses
GSK-3β	GSK-3β	Inhibition prevents mitochondrial dysfunction, apoptosis, and adverse remodeling	SB216763, Tideglusib, Lithium chloride (LiCl)	Promotes cardioprotection, facilitates post-injury recovery	Chronic inhibition may impair metabolic homeostasis and increase fibrosis

## Data Availability

No new data were created.
